# Severe lactic acidosis and persistent diastolic hypotension following standard dose of intermittent nebulized salbutamol in a child: a case report

**DOI:** 10.1186/s13256-022-03357-z

**Published:** 2022-04-22

**Authors:** Marco Colombo, Anna Plebani, Annalisa Bosco, Massimo Agosti

**Affiliations:** 1 Pediatric Emergency Department, ASST Sette Laghi, Ospedale F. del Ponte, Varese, Italy; 2 Department of Neonatology and Pediatrics, ASST Sette Laghi, Ospedale F. del Ponte, Varese, Italy

**Keywords:** Asthma exacerbations, Salbutamol adverse drug reaction, Lactic acidosis, Diastolic hypotension

## Abstract

**Background:**

Salbutamol is a selective β2-receptor agonist widely used to treat asthma in both emergency and outpatient settings. However, it has been associated with a broad spectrum of side effects. Lactic acidosis and diastolic hypotension are rarely reported together following intermittent salbutamol nebulization in children, even less so at standard therapeutic doses.

**Case presentation:**

We present the case of a 12-year-old Italian boy, 34 kg body weight, who experienced a serious drug reaction during a moderate asthma exacerbation with associated dehydration (blood urea nitrogen/creatinine 0.25), following intermittent inhaled (0.2 mg at 3-hour intervals—overall 1.4 mg in 24 hours before arrival) and nebulized treatment (3.25 mg at 20-minute intervals in 60 minutes, overall 11.25 mg in our emergency department). The patient developed hyperglycemia (peak concentration 222 mg/dL), hypokalemia (lowest concentration 2.6 mEq/L), electrocardiogram alterations (corrected QT interval 467 ms), long-lasting arterial hypotension despite fluid boluses (lowest value 87/33 mmHg), and elevated blood lactate levels (peak concentration 8.1 mmol/L), following the third nebulized dose. Infections, liver dysfunction, and toxicity following other medications were ruled out. The aforementioned alterations improved within 24 hours after discontinuation of salbutamol.

**Conclusions:**

We reinforce the message that even the use of intermittent nebulized salbutamol for acute moderate asthma can lead to severe transient complications in children. Then, healthcare providers should pay attention not only in emergency settings, to achieve prompt recognition and proper management of this adverse reaction. Careful reassessment could prevent similar reactions.

## Background

Asthma exacerbation is one of the most common reasons for pediatric emergency department (PED) consultation and hospitalization. Salbutamol is the medication of choice in Italy to treat asthma exacerbation [1]. Both metered dose inhaler with spacer and nebulization are available to administer bronchodilator therapy; the former is preferred for domiciliary use or milder exacerbations, and the latter is preferred for management of more severe exacerbations, mainly in hypoxic and/or noncompliant patients in emergency settings. However, reports of adverse cardiovascular and metabolic drug reactions to salbutamol are increasing. Prompt recognition of these side effects by pediatricians is desirable to prevent bad outcomes and inappropriate treatment.

Herein we describe a serious drug reaction to a standard dose of intermittent nebulized salbutamol in a 12-year-old boy, who required prolonged, intensive observation in our PED.

## Case presentation

A 12-year-old Italian boy, 34 kg body weight, was referred to the PED with a 24-hour history of intermittent chest pain, cough, wheezing, and mild fever. His medications prior to arrival consisted of 0.2 mg of inhaled salbutamol every 3 hours, initiated the previous day without medical advice (overall 1.4 mg/24 hours). The last administration had been performed 1 hour before arrival. No inciting exercise was reported, and use of hypoallergenic material was reported in his bedroom. Salbutamol inhaler had been prescribed by his pneumologist as part of patient’s asthma action plan. Noteworthily, montelukast treatment was discontinued 5 days earlier on the advice of his pneumologist, after a 3-month period without asthma exacerbations.

Past medical history included intermittent asthma induced by exercise and by dust mites diagnosed 1 year earlier. Only one mild exacerbation occurred 9 months following diagnosis, which did not require hospitalization. At that time, hypoallergenic material was not available in his bedroom.

At presentation, vital signs were as follows: heart rate 110 beats per minute, respiratory rate 38 breaths per minute, 94% oxygen saturation on room air, and temperature 37.1 °C.

Physical examination showed intercostal and substernal retractions, diffuse reductions in normal breath sound, and end-expiratory wheezing. His ability to speak was not affected. A pediatric asthma score of 9 was then calculated [2]. Blood gas test results at presentation as well as other blood examination results and vital signs are listed in Table 1.

**Table 1:** Laboratory findings and vital signs

	Arrival	+1 hour Presyncope	+1.5 hours Extreme pallor	+2 hours After two RA boluses	+7 hours Observation	+24 hours Observation	+27 hours Discharge
RR (acts per minute)	38	28	30	24		22	28
HR (beats per minute)	102	140	140	125	140	87	96
BP (mmHg)	110/75	110/80	90/40	87/33	100/40	109/59	116/72
O_2_ sat (%)	94	98	84	100	98	98	97
Temperature (°C)	37.1						36.8
pH	7.41 (a)	7.33 (v)	7.32 (v)	7.38 (a)	7.39 (a)		7.44 (v)
FiO_2_	Room air	Room air	Room air	0.3 Venturi	Room air	Room air	Room air
pO_2_ (mmHg)	66	52	57	63	89		52
pCO_2_ (mmHg)	34	35	33	25	29		35
HCO_3_^−^ (mmol/L)	21.6	18.8	17.4	15.2	17.9		24.2
BE (mmol/L)	− 2.3	− 6.2	− 7.7	− 8.3	− 5.7		+ 0.4
Lactate (mmol/L)	3.9	5.3	5.3	8.1	2.3		0.7
Glucose (mg/dL)	103	213	218	222	123		90
K^+^ (mEq/L)	4.2	2.6	2.9		4.1		3.5
Na^+^ (mEq/L)	141	141	141		138		143
Cl^−^ (mEq/L)	105	109	112		112		109
Hb (g/dL)	15.2	13	12.1	12.8	13.9		12.9
Leukocyte 10^9^/L	6.58						
Creatinine (mg/dL)	0.65						
Urea (mg/dL)	35						
BUN (mg/dL)	16.33						
CRP (mg/dL)	0.32						
AST–ALT (U/L)	24–19						
Troponin-T (ng/mL)			< 0.03				

Empty spaces mean that data are not available

*RR* respiratory rate; *HR* heart rate; *BP* blood pressure; *O*_*2*_* sat* oxygen saturation; *RA* Ringer’s acetate; *v* venous; *a* arterial; *FiO*_*2*_ fraction of inspired oxygen; *pO*_*2*_ oxygen tension; *pCO*_*2*_ carbon dioxide tension; *HCO*_*3*_^*−*^ bicarbonate concentration; *BE* base excess, *BUN* blood urea nitrogen; *CRP* C-reactive protein

The patient was diagnosed with moderate asthma attack and was therefore treated with 40 mg intravenous methylprednisolone and 3.75 mg (0.11 mg/kg) nebulized salbutamol together with 0.5 mg ipratropium bromide at 20-minute intervals. Three oxygen-driven nebulizations were performed because of persistent wheezing, retractions, and moderate hypoxia (oxygen saturation between 90% and 95% on room air).

Five minutes after the end of the third nebulization, a presyncope episode occurred. At onset, vital signs were normal, apart from tachycardia (140 beats per minute); on physical examination, he had tremor in his hands, looked pale, and was eupneic, with normal breath sounds and weak radial pulse. A 12-lead electrocardiogram (ECG) was obtained [aspecific alteration of ventricular repolarization, corrected QT (QTc) interval 467 ms], together with blood samples, showing normal troponin-T serum level. Therefore, a normal saline infusion was started.

Clinical conditions swiftly worsened in the following 30 minutes as the patient suffered faintness, extreme pallor, and cold extremities. His vital signs revealed 84% oxygen saturation on room air, hypotension (90/40 mmHg), and tachycardia (140 beats per minute). A blood gas test was conducted, showing metabolic acidosis with elevated blood lactate levels, hypokalemia, and hyperglycemia. As these findings were consistent with β2-receptor-agonist side effects, salbutamol was discontinued. Consequently, two boluses of Ringer’s acetate were administered, each over 20 minutes, because of persistent hypotension; hypokalemia was treated with 500 mL normal saline and 40 mEq/L of potassium chloride at 70 mL/hour rate of infusion in 3 hours; oxygen supplementation was started with Venturi mask providing a maximum FiO_2_ of 0.3.

The patient was subsequently admitted to our intensive observation unit. During the observation period, the patient remained eupneic without expiratory wheezing. Hypokalemia quickly reverted, whereas lactic acidosis (peak concentration 8.1 mmol/L) and lower DBP persisted for a longer time. Oxygen supplementation was discontinued overnight, maintenance normal saline was continued, and diuresis remained normal. Fluid boluses had been stopped because of persistent lower DBP with normal systolic blood pressure. Ipratropium bromide was administered alone as bronchodilator treatment at 4-hour intervals.

Twenty-four hours later, another 12-lead ECG was performed (sinus rhythm, normal QTc). A pediatric cardiology consultation excluded any evidence of compromised left ventricular function and cardiac output and suggested that the aforementioned ECG alterations might have had a multifactorial origin (mild fever, hypokalemia, and β2-agonist toxicity).

The patient was eventually discharged in good clinical condition with oral betamethasone 1 mg/kg for 3 days and nebulized ipratropium bromide 0.5 mg four times a day. A drug challenge with 0.2 mg of inhaled salbutamol with spacer performed 1 week later was uneventful.

## Discussion and conclusions

This case describes a serious adverse drug reaction (ADR) to salbutamol, as it caused a prolongation of existing hospitalization [3] despite the asthma exacerbation having shown relative swift improvement.

We could not detect any inciting factor and are confident that the patient was given the aforementioned medications prior to admission.

Salbutamol can be administered via spacers or nebulization. Although the use of spacers with metered dose has been shown to be as effective as nebulization to treat asthma exacerbations [4], their use in hospital is very limited in Italy [5]. Despite its deemed safety, reports of ADR are increasing in recent times, accounting for 1310 reports in 10 years from 2006 to 2016 from the European Medicines Agency, EudraVigilance database [6]. However, even if an ADR is reported in 34.6–52% of intermittent salbutamol administrations in children, only 6% of these are thought to be true ADRs, according to Leung *et al.* [7].

Despite its recognized and well-known β2 airway receptor selectivity [8], salbutamol also exhibits β1 activity, especially at higher doses [9], and β3 activity [10], which can lead to a broad spectrum of side effects. Common side effects include tremor [11], dizziness, headache, reduced DBP [12–14], elevated troponin serum level [15], hypokalemia, acute urinary retention [10], and lactic acidosis [16] throughout the stimulation of the β2-adrenergic receptor, which generates aerobic glycolysis leading to hyperglycemia [17] and high tissue concentration of pyruvate, which is eventually converted to lactate by lactate dehydrogenase (type B2 lactic acidosis).

Diastolic hypotension is due to relaxation of vascular smooth muscle that might limit myocardial blood flow, and it was first reported by Shurman *et al.* in 1984 in a 33-year-old man after salbutamol inhalation [18].

Severe side effects are more common following intravenous [19] or oral administration [18], or high-dose continuous nebulized therapy [7]. In addition, corticosteroids may enhance the sensitivity of β-adrenoceptors to sympathetic agents, thus increasing the risk of ADR [20].

Sarnaik *et al.* [12] demonstrated a dose-dependent effect of high-dose continuous inhaled salbutamol on DBP in two cohorts of children with status asthmaticus during transport to the hospital or PICU admission (respectively 56% and 98% of children had at least one episode of low DBP). Fagbuyi *et al.* [15] found elevated troponin-T levels in 25% of children receiving at least 20 μg per hour continuous nebulized salbutamol for a median duration of 40 hours. Carrol *et al.* [13] noticed lower DBP in 66% of children who received 10–15 mg per hour continuous salbutamol for > 2 hours. In addition 15% of the patients were found to have ECG ST-segment change, and 24% had increased troponin-T serum levels. Finally, Wisecup *et al.* [14] found that lower DBP developed in 90% of children receiving a median weight-based dose of continuous nebulized salbutamol of 12.7 mg/kg for status asthmaticus, with a positive correlation with increasing doses.

Severe ADR following intermittent nebulization is rarely reported in younger children, hypothetically because of a less satisfactory nebulizer technique leading to a lower drug absorption [21].

In 2015, Saadia *et al.* [22] first described a 13-year-old female with intermittent asthma who developed lactic acidosis and diastolic hypotension after receiving 22.5 mg of salbutamol intermittent nebulizer treatment, which reverted after drug discontinuation and recurred with drug readministration.

Lactic acidosis is a common finding in poor tissue perfusion or asthma, hypothetically due to inadequate oxygen delivery to the respiratory muscles (type A lactic acidosis) and increased insensible loss due to tachypnea [23]. Moreover, blood urea nitrogen (BUN)/creatine ratio and first blood gas analysis showing a slight increase in lactate and higher level of hemoglobin suggest a possible underlying hypovolemia. In our patient, all these factors might have generated lactic acidosis, whereas salbutamol treatment might have been a contributory aggravating factor.

An increased lactate-to-pyruvate concentration ratio has been deemed a useful tool to distinguish type A and B lactic acidosis [19]; however, its actual utility is controversial [24]. Moreover, we did not utilize it because it was not available at our ED.

Interestingly, our patient experienced also hyperglycemia, tremor, and hypokalemia despite a lower dose than the ones reported in the aforementioned citations. In addition, we did not detect any side effects following the drug challenge performed 1 week later.

Given this, the diagnosis of salbutamol adverse drug reaction may be questionable in our patient; however, dehydration alone cannot explain the contemporary presence of hypokalemia, hyperglycemia, QTc prolongation, and persistent lower diastolic hypotension. Furthermore, no clinical sign of dehydration was present on physical examination. Hence, we considered the diagnosis of isolated dehydration unlikely.

Finally, we applied the “Naranjo’s ADR correlation scale,” obtaining a score of 6, meaning “probable correlation” (> 9 definite ADR, 5–8 probable ADR, 1–4 possible ADR, 0 doubtful ADR) [25]. Additionally, we ruled out other possible causes, including liver dysfunction (normal liver function tests) adverse reaction to other medications (ipratropium bromide is not considered a risk factor for lactic acidosis when administered with salbutamol, according to a recent prospective observational study by Ruman *et al.* [26]) and infections (negative C-reactive protein, self-limited fever). Questions may arise about fever; however, Ylmaz *et al.* [27] were the first to describe a child who experienced fever as a side effect after salbutamol ingestion.

Unfortunately, a toxin drug screen was not performed and no prior ECG was available.

Specifically, we considered these findings as an early ADR following salbutamol nebulization rather than a delayed ADR following medications prior to arrival, for a few reasons: first, most of the side effects of short-acting β2 agonist show tolerance with chronic and repeated use [28]; second, the patient did not report tremor at admission, and potassium and glucose serum levels were normal as well as blood pressure; third, children older than 5 years of age can experience higher plasma concentrations of salbutamol, despite lower dosage [21].

Even if a recent large meta-analysis on lactic acidosis induced by selective β2-adrenoceptor agonists suggests that lactic acidosis is reversible even in patients who continued the drug [24], withholding salbutamol, using an equally effective alternate drug to treat underlying conditions may be pivotal if it does not affect the acute care of the patient. In our patient, we decided to withdraw salbutamol and continue with ipratropium alone as previously reported by Seay *et al.* in a 14 year-old female who experienced severe lactic acidosis after intermittent and continuous salbutamol treatment [29].

Given this, the management of this kind of ADR is purely symptomatic. Hypokalemia should be corrected carefully with frequent assessment of potassium serum levels. It has been suggested that administration of fluid boluses, before continuous nebulized salbutamol, might prevent diastolic hypotension [14]; however, to the best of our knowledge, evidence of the efficacy of such strategy before intermittent nebulized salbutamol is lacking in children. Fluid bolus administration is a possible option for management of salbutamol-induced hypotension, but it may be counterproductive given the increased antidiuretic hormone secretion during acute asthma [30] and the peripheral vasodilation induced by salbutamol, potentially leading to fluid overload.

In conclusion, to the best of our knowledge, we described a severe adverse drug reaction after the lowest dosage ever reported of intermittent nebulized salbutamol in a child presenting with moderate asthma exacerbation and mild dehydration. Careful reassessment is desirable following each nebulization: clinicians should pay attention to otherwise unexplained lactic acidosis and/or persistent lower diastolic blood pressure while treating asthma exacerbations in children, even at standard doses of intermittent nebulized salbutamol, to prevent poor outcome and inappropriate management.

Further research is needed to clarify whether dehydration may increase the risk of ADR following standard doses of nebulized salbutamol.

## Data Availability

The dataset supporting the conclusions of this article is included within the article.

## Abbreviations

PED: Pediatric emergency department
ADR: Adverse drug reaction
DBP: Diastolic blood pressure
SBP: Systolic blood pressure
PICU: Pediatric intensive care unit
